# *Klebsiella pneumoniae* related community-acquired acute lower respiratory infections in CAMBODIA: clinical characteristics and treatment

**Published:** 2011-01-10

**Authors:** Blandine Rammaert, Sophie Goyet, Julien Beauté, Sopheak Hem, Vantha Te, Patrich Lorn Try, Charles Mayaud, Laurence Borand, Philippe Buchy, Bertrand Guillard, Sirenda Vong

**Affiliations:** 1 Epidemiology and Public Health Unit, Institut Pasteur in Cambodia, Phnom Penh, Cambodia; 2 Biomedical Laboratory, Institut Pasteur in Cambodia, Phnom Penh, Cambodia; 3 Pediatric Department, Donkeo Provincial Hospital, Takeo, Cambodia; 4 Pediatric Department, Provincial Hospital, Kampong Cham, Cambodia; 5 Pneumology and Intensive Care Unit, Hôpital Tenon, Paris, France; 6 Virology Unit, Institut Pasteur in Cambodia, Phnom Penh, Cambodia

## 

In many Asian countries, *Klebsiella pneumoniae* is the second pathogen responsible for community-acquired pneumonia and, as other Gram negative bacilli, might produce extended spectrum β-lactamases (ESBL). Still, very little is known about *K. pneumoniae* implication in acute lower respiratory infections (ALRI) in Cambodia. Here we describe the clinical and radiological features of Cambodian patients presenting with community-acquired *K. pneumoniae* ALRI, for which antibiotics and relevant clinical outcomes were recorded. Through ALRI surveillance in 2 provincial hospitals, *K. pneumoniae* was identified on sputum and blood cultures and confirmed by API20E gallery from adult patients with respiratory symptoms≤14 days. Patients with known tuberculosis or immunodepression were excluded. Clinical, radiological and microbiological data were recorded and patient’s outcome was investigated after hospital discharge. A multivariate analysis of risk factors compared *K. pneumoniae*-infected and *Haemophilus influenzae/Streptococcus pneumoniae*-infected patients, 2 of the main ALRI-related pathogens in Cambodia, adjusted for the following variables: sex, tobacco, alcohol intake, cardiovascular disease, chronic lung disease, diabetes, hepatopathy, no prior treatment, hemoptysis, severity.

During April 2007-December 2009, among 3545 patients enrolled in surveillance, 47 *K. pneumoniae* ALRI were diagnosed in sputum (97.8%) and blood (2.1%) cultures, representing 7.7% (n=47/608) of identified bacterial etiology. The median age was 55 years (25-79) and 68.1% were females, including 75% postmenopausal women. Of the 43 available X-rays, 30 showed pneumonia (10 were necrotizing), 2 pleurisies and 11 infections on pulmonary sequelae. Severity was determined in 5 patients, 4 having pneumonia. The main known risk factors were previous medication (42.5%), chronic lung diseases (23.4%) and tobacco (21.3%); 10 patients were co-infected with a virus and 5 with tuberculosis. Producing-ESBL strains were found in 17.0% (n=8/47) of the cases, including 4 in pneumonia cases; most of those being sensitive to ciprofloxacin (n=7/8). An appropriate antibiotherapy according to the antibiogram was given to 13 patients (28%). Overall mortality was 40% (7 lost of-follow-up), higher during hospitalization and within a month after discharge.

When compared with patients presenting with *S. pneumoniae* and *H. influenzae* ALRI, *K pneumoniae* related ALRI were associated with female gender, prior treatment and severity on admission. In conclusion, *K. pneumoniae* related ALRI in Cambodia are often fatal, affect mostly women, and must be considered in patients hospitalized with severity criteria. The frequency of ESBL strains is extremely high among our infected patients. This is alarming in the context of high antibiotics intake often inappropriate and in absence of microbiology capacity in most public-sector hospitals.

